# Geometry Controls Confined Water Dynamics in Lipidic Mesophases

**DOI:** 10.1002/anie.202522757

**Published:** 2026-01-10

**Authors:** Sara Catalini, Matteo Rutsch, Andrea Lapini, Barbara Rossi, Mariangela Di Donato, Brenda Bracco, Marco Paolantoni, Yang Yao

**Affiliations:** ^1^ Department of Chemistry University of Basel Basel Switzerland; ^2^ Department of Chemistry “Ugo Schiff” University of Florence Florence Italy; ^3^ European Laboratory for Non‐Linear Spectroscopy Florence Italy; ^4^ Department of Chemical, Life and Environmental Sustainability Sciences University of Parma Parma Italy; ^5^ Elettra‐Sincrotrone Trieste Trieste Italy; ^6^ CNR‐ICCOM Florence Italy; ^7^ Department of Chemistry Biology and Biotechnology University of Perugia Perugia Italy

**Keywords:** Interfaces, IR spectroscopy, Lipidic mesophases, Phase transitions, Water dynamics

## Abstract

Water under nanoscale confinement is central to biological function, catalysis, and soft materials, yet how geometry dictates its structure and dynamics remains unresolved. Here, we establish a direct link between interfacial curvature and confined water behavior using an archaeal‐inspired phytantriol‐water lipidic mesophase platform. By systematically tuning curvature across lamellar, double‐gyroid cubic, and reverse micellar phases, and integrating structural, thermodynamic, and ultrafast spectroscopies, we show that geometry controls the dimensionality and mobility of the hydrogen‐bond network. Planar interfaces enforce 2D networks that slow down interfacial water through spatial constrain, whereas curved bicontinuous and micellar topologies promote 3D networks with accelerated reorientation. These findings reveal a geometric principle for governing water dynamics in soft nanoconfinement, providing molecular level design rules for confined transport and reactivity in membranes and functional materials.

## Graphical abstract

An archaeal‐inspired lipidic mesophase allows precise control of interfacial curvature and water‐channel connectivity. Combining structural, thermodynamic, and ultrafast spectroscopies, we reveal that curvature governs the structure and reorientation dynamics of confined water, establishing a molecular‐level link between topology and dynamics in soft nanoconfinement.

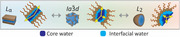

## Introduction

Water plays a crucial role in biological systems, not merely as a solvent, but as an active participant in biochemical processes. Within cells, where macromolecules occupy up to 30% of the total volume,^[^ ^1^ ^]^ water exists in a crowded and chemically heterogeneous environment that profoundly shapes its structure and dynamics through hydrogen bonding, electrostatic interactions and hydrophobic effects.^[^ ^2^ ^]^ As a result, intracellular water differs markedly from bulk water, existing as two main populations: a mobile “core” water and a more tightly bound “interfacial” water closely associated with biomolecular surfaces and exhibiting reduced mobility.^[^ ^3^, ^4^ ^]^

In extremophiles such as archaea, water confinement is modulated by a unique membrane architecture composed of ether‐linked phytanyl chains, which endow the membrane with remarkable stability under extreme thermal and chemical conditions.^[^ ^5^, ^6^ ^]^ Synthetic lipids provide valuable models to explore such effects: monolinolein resembles bacterial lipids, whereas phytantriol captures key structural features of archaeal membranes. When combined with water or other polar molecules, these amphiphiles spontaneously self‐assemble into lipidic mesophases (LMPs)^[^ ^7^, ^8^, ^9^, ^10^ ^]^ whose structures depend sensitively on temperature and hydration.^[^ ^11^, ^12^ ^]^

In phytantriol‐water systems, a series of well‐defined phase transitions occur from lamellar (*L*_α_) to reverse micellar (*L*_2_) structures through the intermediate double gyroid cubic phase ($Ia\bar{3}d$). These lipidic mesophases, which serve as versatile model systems in biology and advanced materials design,^[^ ^13^ ^]^ present nanoscale water compartments that enable the selective transport of molecules,^[^ ^14^ ^]^ molecular separation, catalysis, and energy storage.^[^ ^7^, ^8^, ^15^ ^]^ The *L*_α_ phase consists of stacked lipid bilayers in which hydrophilic headgroups face the aqueous environment and hydrophobic tails align inward, closely resembling cellular membranes.^[^ ^16^ ^]^ In contrast, the $Ia\bar{3}d$ phase contains two interpenetrating but non‐communicating water channels, separated by a continuous lipid bilayer that smoothly bends in three dimensions. Such cubic phases occur in living systems, for instance during membrane remodeling and in specialized organelles,^[^ ^17^ ^]^ and are exploited in advanced materials such as photonic crystals,^[^ ^18^, ^19^ ^]^ underscoring their functional significance. Reverse micelles, on the other hand, arise when lipid headgroups orient inward to encapsulate small water pools, while hydrophobic tails extend outward into a nonpolar medium. These structures resemble intracellular vesicles or lipid droplets, where polar solutes are sequestered within a hydrophobic matrix.^[^ ^20^ ^]^

The distinct topologies of these mesophases arise from variations in lipid tail packing and interfacial curvature, which together determine the architecture of the confined water network.^[^ ^21^ ^]^ In the *L*_α_ structures, water forms flat, 2D sheets confined between bilayers, while in the $Ia\bar{3}d$ phase, water occupies the continuous 3D network. In the *L*_2_ phase, water is compartmentalized into discrete droplets enclosed by a lipid monolayer; although isolated, the water within these droplets still maintains a 3D hydrogen‐bonded network. In small micelles, the high curvature and restricted volume strongly perturb hydrogen‐bonding, altering both the structure and dynamics of the confined water.^[^ ^22^ ^]^ These differences are not merely morphological but fundamentally affect the dynamics, reactivity, and stability of biomolecules, with broad implications for biochemical processes in crowded or confined environments.^[^ ^23^ ^]^

The transition from continuous water networks to discrete water droplets provides a structural framework for understanding how soft confinement governs water dynamics and molecular organization. From a materials science perspective, such topological changes offer a design handle for modulating molecular transport and release:^[^ ^24^ ^]^ continuous phases support long‐range diffusion and connectivity, whereas discrete micellar domains favor encapsulation and retention. This geometry–transport relationship is directly relevant to the rational design of lipid‐based functional materials, including controlled drug delivery matrices and responsive nanoscale architectures.^[^ ^25^ ^]^

Here, we investigate the general principle that the behavior of confined water in soft matter is dictated not only by the degree of confinement but also by the geometry of the confining interface, specifically its curvature, continuity, and the dimensionality of the accessible water network. We propose two hypotheses: (i) changes in mesophase topology reorganize the hydrogen‐bond network in discrete steps, producing distinct interfacial and core water populations with characteristic dynamics, and (ii) the continuity of the water network imprints a measurable signature on lipid packing and charge transport. To test these hypotheses, we use a single archaeal‐mimetic phytantriol‐water platform in which curvature, aqueous continuity, and dimensionality can be tuned in a controlled manner across *L*_α_, $Ia\bar{3}d$, and *L*_2_ topologies. In our multi‐technique approach, each method addresses a specific mechanistic question. Small‐angle X‐ray scattering (SAXS) defines the mesophase geometry, curvature, and water channel diameter. Vibrational spectroscopy (Fourier transform infrared (FTIR) and ultraviolet resonance Raman (UVRR)) probes the hydrogen‐bond populations and interfacial hydration, providing global and site‐specific structural insights. Ultrafast IR spectroscopy^[^ ^26^, ^27^ ^]^ resolves picosecond reorientation of water and distinguishes between 2D and 3D water network behavior. Calorimetric and dielectric measurements reveal how geometric transitions couple to phase energetics and the electrodynamic response. Together, these complementary probes allow us to extract a unified framework in which mesophase geometry prescribes the organization, mobility, and connectivity of confined water.

## Results and Discussion

**Figure 1:**
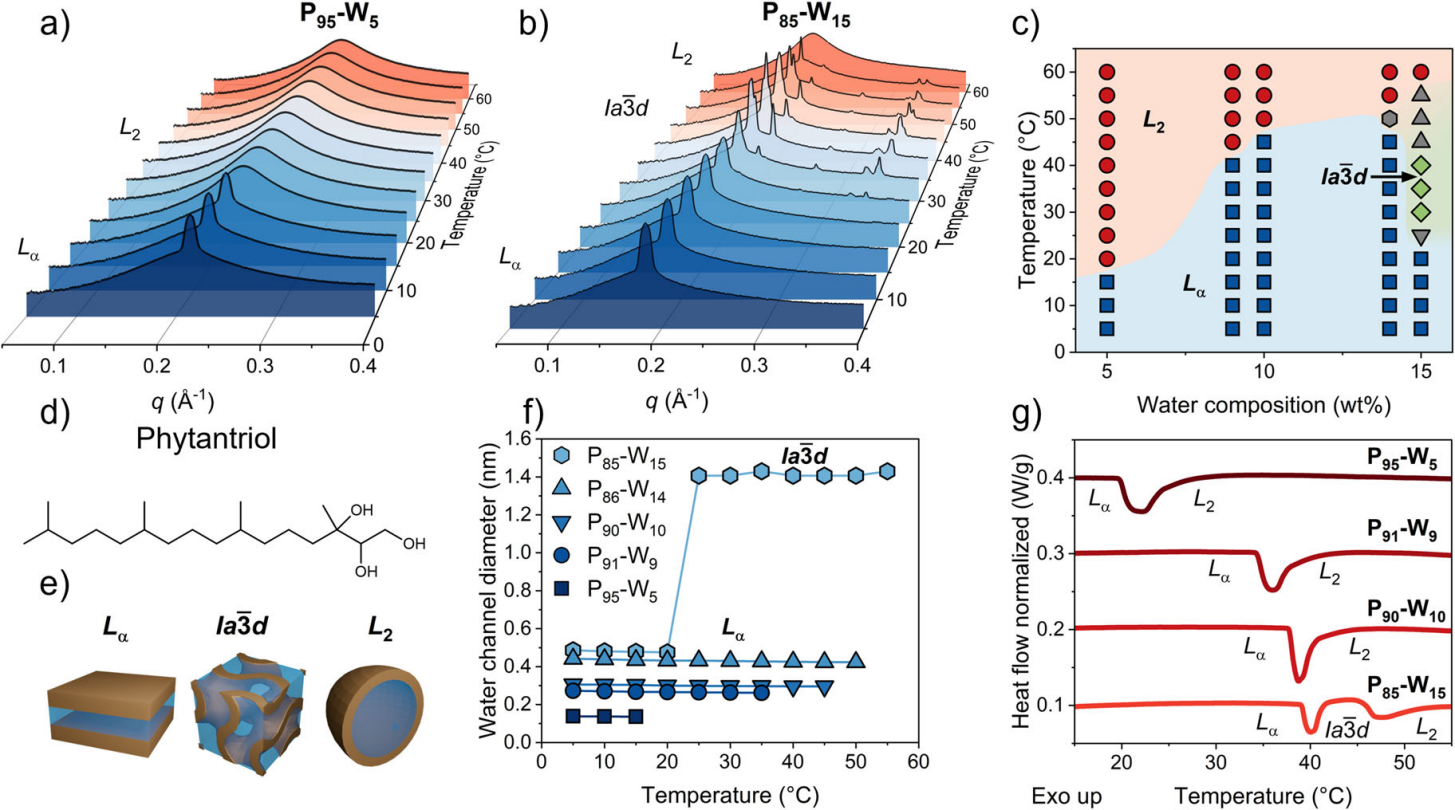
SAXS profiles of LMPs containing 5 wt% a), and 15 wt% b) water. c) Phase diagram of the phytantriol‐water LMPs as a function of water composition and temperature obtained by SAXS. Grey symbols correspond to mixed symmetries. d) Molecular structure of phytantriol. e) Schematics of the *L*_α_, $Ia\bar{3}d$, and *L*_2_ phase structures. f) Calculated water channel diameter of LMPs with different water content based on the structural parameters obtained by SAXS. Error bars are smaller than the symbols. g) Representative DSC heating curves for phytantriol‐water LMPs with different water fraction recorded at 10 °C min^−1^ rate, the curves were vertically shifted for better visibility.

The phase structures of phytantriol‐water mixtures were characterized by SAXS (Figure 1). The representative SAXS profiles of the 5 wt% (P_95_‐W_5_) and 15 wt% (P_85_‐W_15_) water samples are shown in Figure 1a,b, and data for 9 wt% (P_91_‐W_9_), 10 wt% (P_90_‐W_10_) and 14 wt% (P_86_‐W_14_) are provided in Figure S1a–c. All the samples show distinct LMP structures depending on the water content and the temperature, as summarized in the phase diagram in Figure 1c. The molecular structure of phytantriol and schematics of the different LMPs are shown in Figure 1d,e, respectively. The phase behavior is consistent with previous studies on the phytantriol‐water system,^[^ ^12^ ^]^ though some variation arises, depending on the batch differences of phytantriol.^[^ ^28^ ^]^

At low temperatures (5 °C–15 °C) all samples present a lamellar phase (*L*_α_), identified by a sharp diffraction peak at a characteristic wavevector, *q*, corresponding to the bilayer spacing (Figure 1a,b). Upon heating, all the samples experience a phase transition to a reverse micellar phase (*L*_2_) at a temperature that depended on the water content (Figure 1c). The SAXS profile of *L*_2_ phase shows a single broad peak that corresponds to the average distance between reverse micelles and the absence of well‐defined long‐range packing of lipids (Figure 1a,b). Notably, the 15 wt% water sample exhibits different Bragg reflections between 25 °C and 55 °C with relative positions in ratios $\sqrt{6}:\sqrt{8}:\sqrt{14}:\sqrt{16}:\sqrt{20}:\sqrt{22}$, which reflects the bicontinuous cubic phase with $Ia\bar{3}d$ symmetry (Figure 1b). The sequence of phase transitions is consistent with general lipid‐water self‐assembly behavior. Increasing temperature enhances the mobility of the hydrophobic tails and their occupied volume, favoring phase structures with higher curvature.^[^ ^29^ ^]^ On the other hand, increasing water content results in larger headgroup volume due to hydrogen bonding interaction between water and lipid heads, thereby stabilizing phase structures with lower curvature.^[^ ^10^ ^]^

The diameter of water domains confined in LMPs has been calculated based on the structural parameters of the *L*_α_ and $Ia\bar{3}d$ phases as a function of temperature, as detailed in the Supporting Information. Figure 1f reports the temperature dependence of the water channel diameter. Within the same phase, the water channel diameter decreases slightly with increasing temperature but increases markedly with higher water content. At 5 °C, increasing the water content from 5 wt% to 15 wt% leads to swelling of the lamellar spacing by ∼0.35 nm. In the 15 wt% water LMP, during the phase transition from *L*_α_ to $Ia\bar{3}d$, the aqueous domain expands by ∼0.8 nm. This transition is accompanied by a structural change from a discontinuous, 2D water arrangement in the *L*_α_ phase to a continuous, 3D water network in the $Ia\bar{3}d$ phase. These observations highlight the critical role of phase geometry in the connectivity of water molecules within lipidic mesophases.

The thermodynamics of the phytantriol–water system during phase transitions was studied by differential scanning calorimetry (DSC). Phase transition temperatures and enthalpy changes were determined during both cooling and heating cycles (Figure S2). All transitions observed by SAXS were also detected by DSC, with only minor temperature deviations attributed to the different measurement modes, i.e., SAXS being isothermal and DSC isochronal. Due to the kinetic nature of phase transitions in LMPs, the transition temperatures differ slightly between cooling and heating cycles. This is particularly evident in the heating‐rate dependence of the transition temperatures, as shown in Figure S2c,f. Representative DSC curves recorded at a heating and cooling rate of 10 °C/min are presented in Figure 1g. These results clearly show that the *L*_2_ → *L*_α_ transition temperature increases with increasing water content. The corresponding enthalpy changes were also quantified. These reflect the energetic cost of lipid reorganization, particularly the formation of bilayer structures during the transition from the disordered *L*_2_ phase to either the lamellar *L*_α_ or the cubic $Ia\bar{3}d$ phase upon cooling. The *L*_2_ → *L*_α_ transition exhibited relatively low enthalpy changes (0.75–1.12 J/g), whereas the *L*_2_ → $Ia\bar{3}d$ and $Ia\bar{3}d$ → *L*_α_ transitions showed even smaller values (0.45–0.67 J/g). Interestingly, the sum of the enthalpy values for the *L*_2_ → $Ia\bar{3}d$ and $Ia\bar{3}d$ → *L*_α_ transitions closely matches that of the direct *L*_2_ → *L*_α_ transition (Figure S2b,e), suggesting comparable overall energetic requirements. Overall, these values are in line with those previously reported for equivalent phase transitions in comparable LMP systems.^[^ ^30^ ^]^

**Figure 2:**
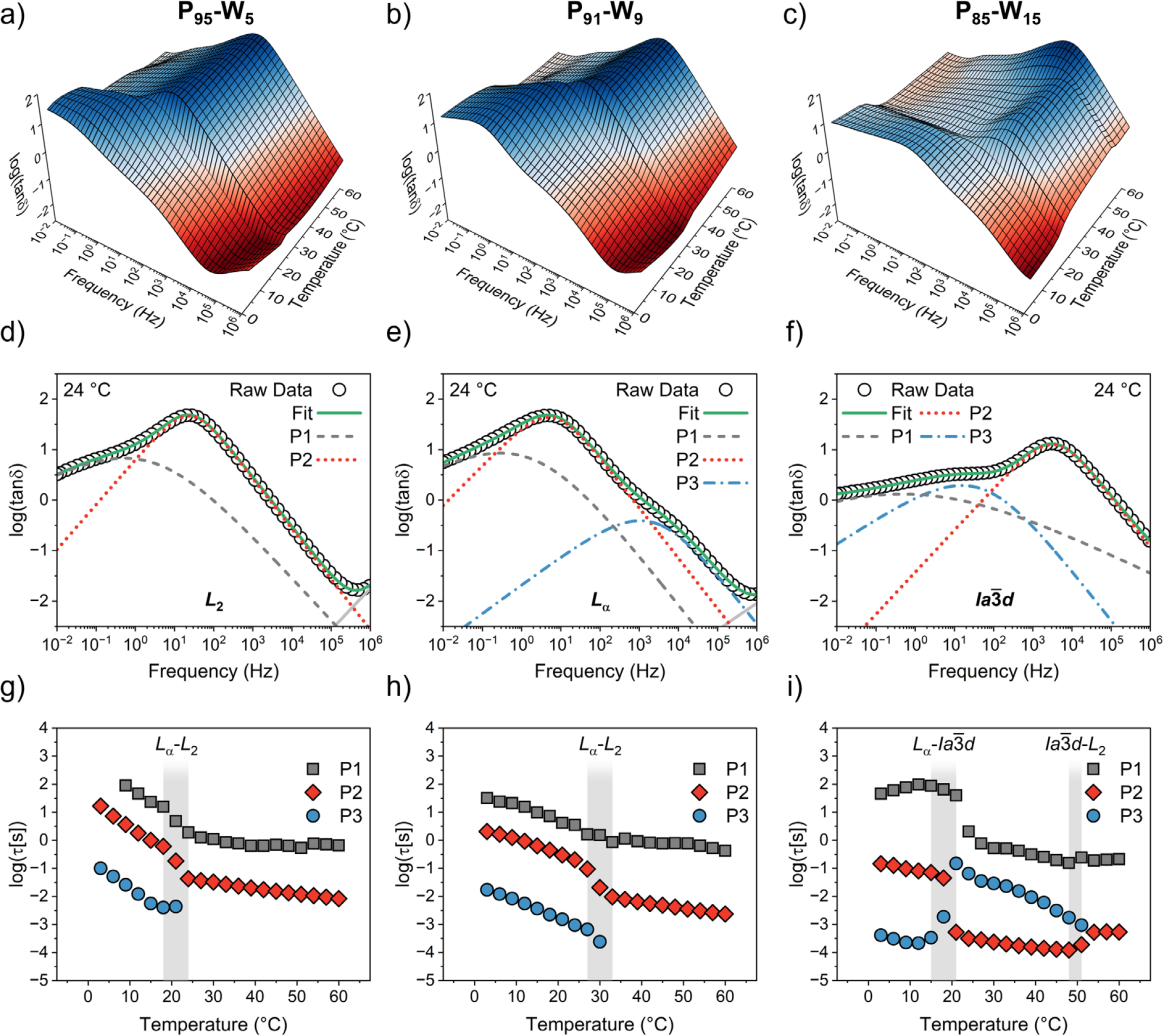
3D representation of tanδ as a function of frequency in the 3–60 °C temperature range for LMPs with 5 wt% a), 9 wt% b) and 15 wt% water c). Fitting examples for LMPs at 24 °C with 5 wt% d), 9 wt% e) and 15 wt% f) water, representative of the three different topologies *L*_2_, *L*_α_ and $Ia\bar{3}d$ phases, respectively. The light grey line corresponds to relaxation processes that lie partially outside the observed frequency window. Relaxation times obtained from Havriliak‐Negami (HN) fittings of BDS tanδ spectra for LMPs with 5 wt% g), 9 wt% h) and 15 wt% i) water. The grey shaded areas indicate the phase transitions with the corresponding phase symmetries.

To investigate the lipid dynamics in our LMPs during the phase transitions, we performed isothermal broadband dielectric spectroscopy (BDS) measurements over a frequency range of 10^−2^–10^6^ Hz and a temperature range of 3–60 °C. The 3D plots of tan δ ($\tan\delta =\frac{\varepsilon^{\prime \prime }}{\varepsilon^{\prime }}$) (Figure 2a–c) show distinct kinks at phase transition temperatures that were previously identified by SAXS and DSC. The spectrum at each temperature was fitted with up to three relaxation processes. Representative fits for LMPs containing 5 wt%, 9 wt%, and 15 wt% water at 24 °C exhibiting *L*_2_, *L*_α_, and $Ia\bar{3}d$ phase, respectively, are shown in Figure 2d–f. The corresponding relaxation times, with the phase transition indicated by the grey area, are shown in Figure 2g–i. The summary of fitting parameters is shown in Figure S3a–f.

The slowest process, P1, is attributed to electrode polarization, associated with accumulation of charges at the sample–electrode interface. This process relates to the formation of an electric double layer, leading to a decrease in the real part of the complex conductivity (σʹ), observed for all LMPs (Figure S5).^[^ ^31^ ^]^ The intermediate process P2 exhibits a Debye‐like distribution (α ≈ 1 and αγ  =  1) with relaxation times (τ_σ_) aligning with the crossover of the real and imaginary part of the complex electric modulus M* (see Figure S3g–i), confirming its assignment to conductivity relaxation (also called electrical relaxation).^[^ ^32^ ^]^ The conductivity relaxation is related to the frequency‐dependent response of charge carriers. In a simple homogeneous system, conductivity relaxation accelerates with an increase in temperature (i.e., τ_σ_ decreases). In contrast, our LMPs consistently exhibit a kink in τ_σ_ at phase transitions, indicating that conductivity relaxation is governed by the underlying phase structure. The τ_σ_ decreases during the *L*_α_ → *L*_2_ phase transition (Figure 2g,h), because of the largely reduced viscosity of the *L*_2_ phase.^[^ ^33^ ^]^ However, phase structure dependent viscosity alone is not sufficient to dictate conductivity relaxation. For instance, although $Ia\bar{3}d$ has a much higher viscosity than *L*_α_ phase, τ_σ_ decreases by roughly 2.1 decades during the *L*_α_ → $Ia\bar{3}d$ phase transition. This is due to the formation of two independent and interpenetrating networks of water channels in the bicontinuous mesophase, as revealed by SAXS (Figure 1c,e).^[^ ^33^ ^]^ Upon transition from $Ia\bar{3}d$ to the *L*_2_ phase, τ_σ_ increases mildly, by about 0.6 decades, despite the dramatic viscosity drop in the *L*_2_ phase. This arises from the loss of water‐channel continuity and the formation of reverse micelles, where water is encapsulated by a surrounding lipid layer.

The fastest process in BDS, P3, follows a Cole‐Cole distribution (α < 1 and αγ  =  1) and is observed only in *L*_α_ and $Ia\bar{3}d$ phases (Figure 2g–i). P3 reflects dipole‐matrix interaction, i.e., a collective orientational dynamics of polar headgroups within the LMP matrix.^[^ ^34^ ^]^ The *L*_α_ phase exhibits 1D periodicity due to the regular stacking of lipid bilayers, whereas the cubic $Ia\bar{3}d$ phase displays a 3D periodic lattice, characteristic of its double gyroid structure.^[^ ^35^ ^]^ In contrast, P3 is absent in the *L*_2_ phase, which lacks lattice periodicity and consists of dispersed reverse micelles in a structurally isotropic medium. The dynamics of P3 depend both on the macroscopic mobility of the mesophase and the packing of lipid headgroups. In $Ia\bar{3}d$ phase, tighter headgroup packing and a significantly higher viscosity result in a more rigid lipid interface,^[^ ^36^ ^]^ which explains the slowdown of the dipole‐matrix dynamics during the *L*_α_ →$\;Ia\bar{3}d$ phase transition. P3 was also observed in the *L*_α_ phase at subzero temperatures.^[^ ^12^ ^]^ Figure S4 shows that its relaxation time evolves continuously across the full temperature range. Notably, all relaxation processes observed in this study are significantly slower than both the side‐chain motions (β‐relaxation) and the segmental dynamics (α‐relaxation) of phytantriol (Figure S4).

**Figure 3:**
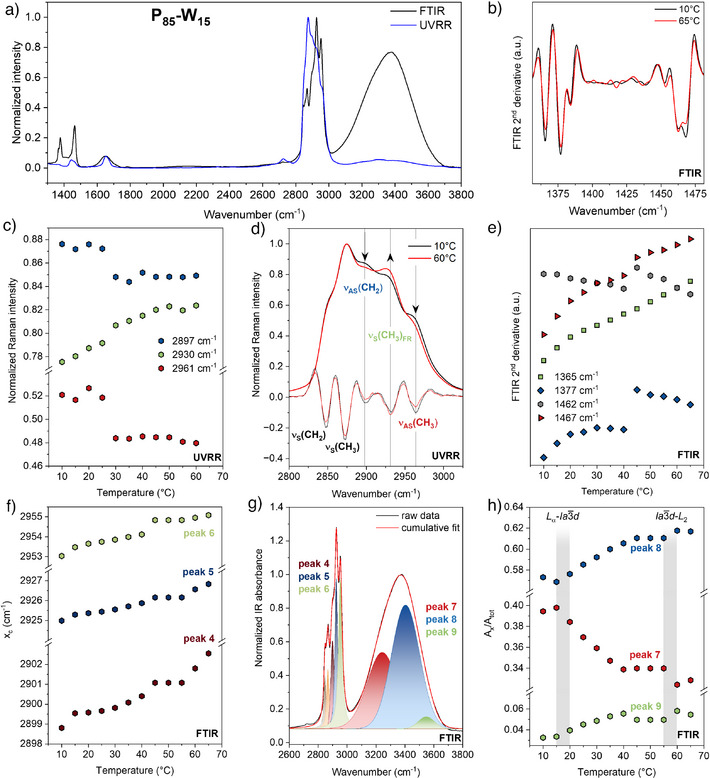
a) FTIR and UVRR spectra of 15 wt% water LMP recorded at 10 °C. b) Second derivative of the FTIR spectra at 10 °C and 65 °C. c) Temperature dependent Raman intensities of the peaks indicated by arrows in panel (d). d) UVRR spectra at 10 °C and 60 °C, with corresponding second derivatives of the spectra. e) Temperature dependence of FTIR spectra second derivative peak intensities. f) Temperature dependence of the peaks position in the lipid region, obtained from FTIR spectral fitting. g) Fitting example of the FTIR spectrum in the 2600–3800 cm^−1^ region at 10 °C. h) Temperature‐dependent fractions of highly ordered, partially disrupted and weakly stabilized OH stretching components derived from the fitting of the FTIR spectra of the 15 wt% water sample. The grey shaded areas indicate the phase transitions with the corresponding phase symmetries.

To gain a comprehensive molecular‐level understanding of phytantriol–water organization, hydration, and structural changes during phase transitions, we combined FTIR and UVRR vibrational spectroscopy. FTIR provides global structural information, including lipid chain order, headgroup hydration, and water hydrogen‐bonding, whereas UVRR offers site‐specific sensitivity to selected molecular groups and their local environments. In this study, deep‐UV excitation at 213 nm enhances Raman bands arising from the lipid hydrocarbon tails thanks to the pre‐resonance effect with σ orbitals (< 200 nm). Although the Raman features of lipids are well studied,^[^ ^37^ ^]^ UV resonance enhancement of lipids remains largely unexplored, and we present here the first UVRR spectra of the lipidic mesophase system. Figure 3a shows the representative FTIR and UVRR spectra of 15 wt% water LMP in both the mid‐frequency (1000–1700 cm^−1^) and high‐frequency (2600–3800 cm^−1^) regions, results for 5 wt% and 10 wt% samples are given in Figures S6 and S7, respectively.

The phytantriol molecule presents two main structural components: the phytyl chain and the triol group (Figure 1d). Vibrations involving CC, CH_2_, and CH_3_ groups of the phytyl chain, as well as the COH groups of the triol moiety, are strongly coupled. Methylene deformation bands (1300–1600 cm^−1^) serve as sensitive indicators of intermolecular interactions between alkyl chains.^[^ ^38^ ^]^ Strong Raman bands at 2850 cm^−1^ and 2870 cm^−1^ correspond to the symmetric stretching vibrations of methylene and methyl groups, respectively (Figure 3d).^[^ ^37^, ^39^ ^]^ The associated asymmetric stretching modes appear at 2897 cm^−1^ and 2961 cm^−1^. A third prominent band near 2930 cm^−1^ is assigned to the coupling between the CH_2_ asymmetric stretching vibration and an overtone of the CH_2_ bending mode through a Fermi resonance (Figure 3d).^[^ ^40^ ^]^ Although this complicates the spectral interpretation, the Fermi resonance provides insight into the local environment and conformational state of methyl groups, with its strength reflecting intramolecular interactions and vibrational coupling.^[^ ^40^ ^]^

UVRR spectra clearly reveal structural differences between phases as reflected by the changes in the relative intensity of the peaks.^[^ ^41^ ^]^ In the *L*_α_ phase, lipid chains are well‐aligned, leading to stronger intensities arising from collective, ordered arrangements that enhance polarizability changes. In contrast, in the *L*_2_ phase, lipid tails are significantly more disordered, adopting a broader range of orientations and conformations. This loss of long‐range order reduces the coherence of polarizability tensor changes, thereby diminishing the 2897 cm^−1^ and 2961 cm^−1^ vibrational bands intensity.^[^ ^41^ ^]^ Meanwhile, the Fermi resonance band near 2930 cm^−1^ increases its intensity upon transition to the *L*_2_ phase, reflecting increased chain disorder and mobility (Figure 3c). In disordered environments, the likelihood of vibrational coupling rises, thereby enhancing the resonance band intensity. This effect is commonly observed when lipid chains move from a highly ordered (*L*_α_, and $Ia\bar{3}d$) to a more fluid and disordered (*L*_2_) phase, where an increase in gauche conformations and a dynamic molecular environment facilitate stronger vibrational coupling.

FTIR spectra show complementary results: peak positions associated with carbon‐chain vibrations (peak 4, 5, 6 in Figure 3f) shift by ca. 2–3 cm^−1^ toward higher wavenumbers as temperature increases, consistent with enhanced tail mobility as the rotation around the CC bonds becomes more favorable, promoting the formation of gauche conformers over trans.^[^ ^42^ ^]^ To better resolve the individual spectral components, the second derivative curves of the FTIR spectra in the low‐frequency region, associated with CH_2_ and CH_3_ deformations, were analyzed (Figure 3b). Tracking peak intensities as a function of temperature (Figure 3e) highlights clear discontinuities at phase transitions, in agreement with high‐frequency region CH_2_ and CH_3_ stretching. Together, these observations indicate reduced carbon chain packing and increased molecular mobility as the system evolves from bilayer (*L*_α_, and $Ia\bar{3}d$) to non‐bilayer (*L*_2_) structure.

The static structure of the hydrogen‐bond network in confined water domain is probed through analysis of the OH stretching band in the 3000–3800 cm^−1^ region (Figure 3g). Hydrogen‐bond strength is inversely correlated with frequency, i.e., more strongly hydrogen‐bonded water molecules exhibit lower OH stretching frequencies. To capture structural variations in the hydrogen‐bond network, the OH stretching band is deconvoluted into three Voigt components corresponding to distinct OH configurations that coexist in dynamic equilibrium, continuously interconverting during transient hydrogen‐bond reorganization. The low‐frequency component (∼3200 cm^−1^) is associated with a highly ordered, tetrahedrally coordinated hydrogen‐bond network. The intermediate (∼3400 cm^−1^) corresponds to partially disrupted hydrogen‐bonds, while the high‐frequency component (∼3600 cm^−1^) represents weakly stabilized OH groups. The relative populations of these configurations vary with both temperature and the topology of the LMP, as previously reported for the monolinolein LMP system.^[^ ^9^, ^43^ ^]^

In bulk water, increasing temperature enhances thermal motion, which progressively disrupts the tetrahedral structure of the hydrogen‐bond network. As a result, the intensity of the 3200 cm^−1^ component decreases, while the intensity of the 3400 cm^−1^ and ca. 3600 cm^−1^ components correspondingly increase, reflecting a transition toward a less ordered water network upon heating. In LMPs, lipid headgroups contribute directly to the OH stretching band through interactions with surrounding water molecules, thereby influencing the spectral profile. The impact of phase transitions on the water network, moving from the *L*_α_ phase, characterized by a 2D water arrangement toward the $Ia\bar{3}d$ and *L*_2_ phases showing 3D water network, can be tracked by reporting the areas of the different hydrogen‐bonding components relative to the total OH stretching band area (Figure 3h). Each OH component exhibits a linear dependence with temperature within a given phase, however, discontinuities emerge at the phase transition temperatures, reflecting an overall structural rearrangement of the hydrogen‐bond network induced by changes in the lipid geometry. The fraction of the area corresponding to the tetrahedrally coordinated hydrogen‐bond network decreases almost linearly from 5 °C to 40 °C, while the fraction of disrupted or weakly stabilized hydrogen bonds increases accordingly. This trend is pronounced up to 40 °C, whereas between 40–60 °C the hydrogen‐bond structure appears largely insensitive to temperature. The transition from *L*_α_ to $Ia\bar{3}d$ to *L*_2_ phases decreases the interfacial area per lipid headgroup, leaving less space for water molecules to interact with the hydroxyl groups of phytantriol, while simultaneously increasing the surface curvature. High curvature strongly disrupts the formation of an ordered network, emphasizing how the thermal response of water depends on the geometry of confinement. Upon transition from the *L*_α_ phase to the $Ia\bar{3}d$ and *L*_2_ phases, the H‐bond network changes from 2D to 3D topology, but confinement and high curvature prevent the formation of an ordered 3D water network as seen in bulk water, explaining the minimal temperature dependence of H‐bonds between 40–60 °C. The weakened temperature dependence of the OH‐stretching band area may also arise from the formation of reverse micelles, which disrupt the continuity of aqueous channels and thereby hinder efficient thermal transport.

**Figure 4:**
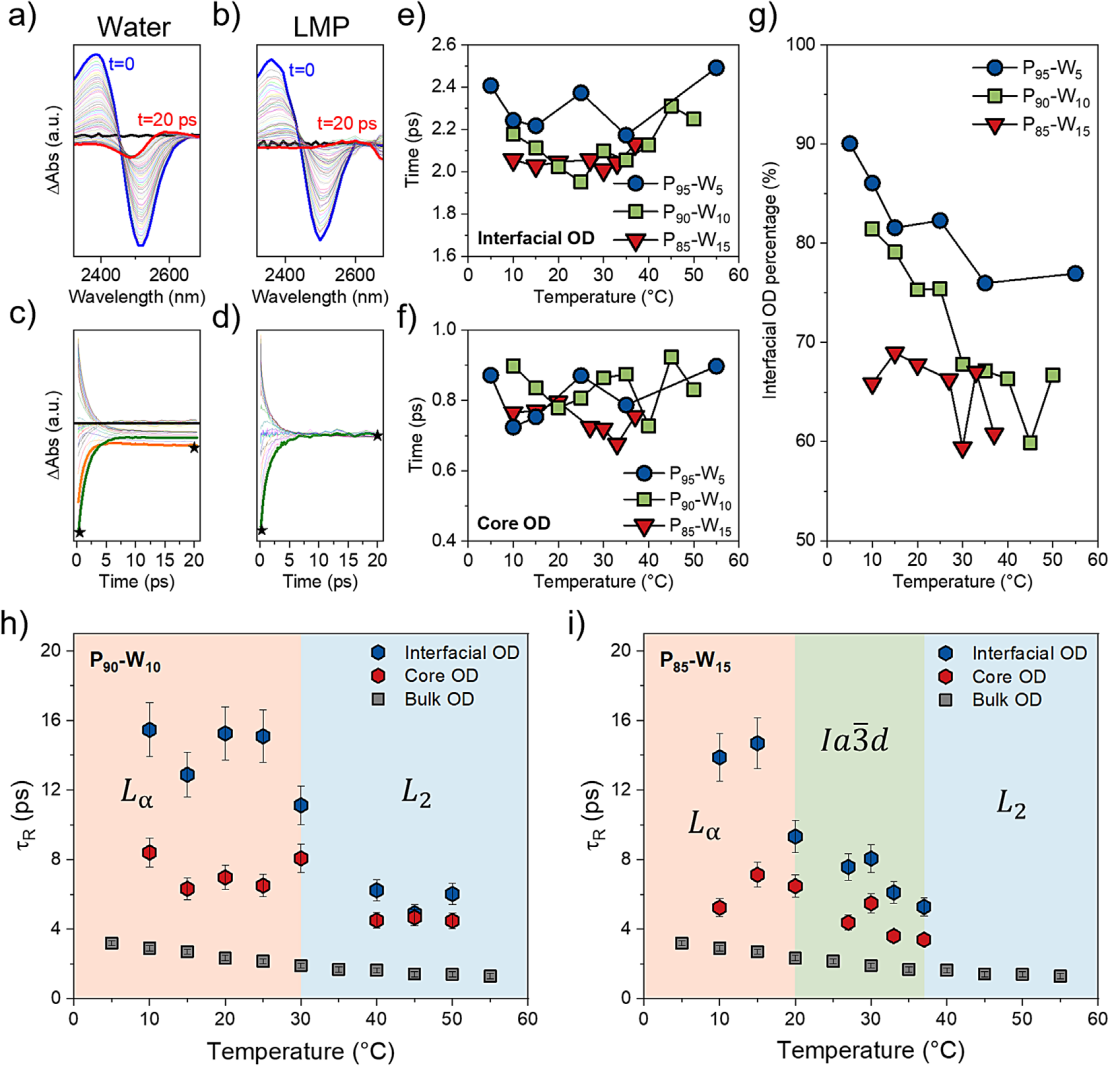
a, b) The transient absorption spectra of pure water and LMP, respectively. Blue spectra are recorded at t = 0 while red spectra at t = 20 ps. c, d) the ΔAbs evolution as a function of time at fixed frequency for pure water and LMP, respectively. e, f) the vibrational relaxation times as a function of temperature of the two components of the transient signal for all the investigated samples. g) the estimated percentage of bound water with the thermal residue method. h, i) the reorientation times of water molecules obtained from pump‐probe experiments for LMP 10 wt% and 15 wt% water as a function of temperature, respectively.

We next investigated the dynamics of confined water using ultrafast pump‐probe IR spectroscopy in the OD stretching region (Figure 4). In this measurement, an intense pump pulse selectively excites the OD stretch in HOD molecules, and a delayed probe pulse tracks the resulting transient absorption (details provided in the Supporting Information). This approach captures the dynamics of hydrogen‐bond rearrangements on picosecond timescales, complementing the static structural insights from FTIR. Representative transient spectra for bulk water and the phytantriol–water LMP are shown in Figure 4a,b, with corresponding kinetic traces in Figure 4c,d.

The temporal evolution of the spectra was fitted with a two‐component model (Figure S8), to extract the excited‐state decay constant associated with vibrational energy dissipation, and to evaluate the presence of multiple relaxation processes.^[^ ^44^ ^]^ This model assumes the presence of two distinct populations of OD oscillators, each vibrating at slightly different frequencies and exhibiting different relaxation dynamics.^[^ ^44^ ^]^ In our system, OD oscillators originate from core water, interfacial water, and phytantriol polar headgroups. Because hydrogen exchange occurs among D_2_O, H_2_O, and phytantriol, all three subpopulations contribute to the measured response. For simplicity and model feasibility, we group interfacial and headgroup‐associated OD oscillators into a single high‐frequency component while assigning the low‐frequency component to core water OD oscillators, reflecting their distinct spectral signatures and environments.^[^ ^45^, ^46^, ^47^, ^48^ ^]^

In bulk water, OD relaxation proceeds via a primary 1.8 ps decay followed by a much slower decay on the order of hundreds of nanoseconds.^[^ ^49^ ^]^ Most of the energy deposited by the pump pulse is rapidly dissipated through vibrational, rotational, and orientational motions. However, a small fraction remains as thermal energy, generating a persistent “thermal residue” on longer timescales. Since this relaxation occurs beyond the 20 ps detection window of our experiment, it appears as a constant offset in the transient spectra. This thermal contribution arises from the increased number of broken intermolecular hydrogen bonds following photoexcitation. The intensity of the thermal signal at 20 ps, relative to the signal at time zero (ΔAbs_(t  =  20 ps)_/ΔAbs_(t  =  0)_), depends on the water content of the sample. For bulk water, this ratio, namely n_water_, is typically ca. 0.3 at room temperature and shows only weak temperature dependence (Figure S9). Thus, the ratio provides an estimate of the residual thermal background arising from bulk water, while the same ratio calculated from the transient signals of the phybtantriol mesophase samples, namely n_sample_, represents the equivalent contribution for water under confinement. Assuming that the thermal residue originates exclusively from bulk‐like water, the ratio n_sample_/n_water_ reported in Equation S6 enables us to estimate the fraction of interfacial water present in the sample.

Across all samples, the extracted excited‐state relaxation times (Figure 4e,f) show that the high‐frequency (interfacial) OD population relaxes in ∼2.0 ps (Figure 4e), whereas the low‐frequency (core) OD relaxes faster, in ∼1.0 ps (Figure 4f), in agreement with previous results obtained for 0.2 nm sodium bis(2‐ethylhexyl) sulfosuccinate (AOT) reverse micelles, reported by Bakker et al.^[^ ^45^ ^]^ The ΔAbs spectra versus frequency (Figure 4a,b) and time (Figure 4c,d) highlight both the spectral signature (red spectra at 20 ps) and temporal persistence (plateau at 20 ps) of the thermal residue. The ratio ΔAbs_(t  =  20 ps)_/ΔAbs_(t  =  0)_ (see Supporting Information) estimates the residual background due to the thermal effect, which is ca. 0.3 for bulk water (Figure S9). In LMPs, the amount of thermal residue with respect to the signal at zero delay differs from that of bulk water, becoming progressively less pronounced as the water content decreases. This trend reflects the greater efficiency of phytantriol and bound water molecules in dissipating energy via vibrational relaxation as compared with bulk water.

Our results suggest that, in LMPs, the thermal residue is only observed for the core water population. Accordingly, the relative weight of the thermal residue component serves as an indirect measure of the portion of interfacial water, as plotted in Figure 4g, estimated using Equation S7. The results reveal a decreasing interfacial water fraction with increasing total water content. Moreover, the fraction of interfacial water also tends to decrease with increasing temperature, as observed in the 5 wt% and 10 wt% samples. The reorientation times of water molecules, τ_R_, extracted from pump–probe measurements using Equation S8, are shown in Figure 4g,h for LMPs containing 10 wt% and 15 wt% water, respectively, with bulk water included for comparison. A clear frequency dependence is observed: blue points in Figure 4h,i correspond to the high‐frequency component (∼2525 cm^−1^, mainly interfacial OD oscillators), and red points to the low‐frequency component (∼2495 cm^−1^, mainly core OD oscillators).^[^ ^45^ ^]^ In both samples, core water consistently reorients faster (shorter τ_R_) than interfacial water, and both populations are significantly slower than bulk water. This behavior reflects differences in intermolecular interactions and geometry: bulk water reorients readily because hydrogen‐bond breaking is rapidly compensated by new bond formation.^[^ ^21^, ^22^ ^]^ A similar mechanism operates for core water, although its motion is restricted within the channels or sheets of the lipid mesophases, leading to slower dynamics. In contrast, interfacial water reorients most slowly, as the reduced number of neighboring molecules and stronger interactions with lipid headgroups hinder its mobility.

In bulk water, τ_R_ decreases steadily with increasing temperature, from 3.0 ps at 5 °C to 1.3 ps at 60 °C. In contrast, τ_R_ in LMPs is highly dependent on phase structure, especially for interfacial water. In the 10 wt% water sample, the τ_R_ of core water decreases from ∼7 ps in the *L*_α_ phase (20 °C) to ∼5 ps in the *L*_2_ phase (50 °C). During the *L*_α_ → *L*_2_ phase transition in 10 wt% water and the *L*_α_ →$\;Ia\bar{3}d$ phase transition in 15 wt% water, interfacial water shows a pronounced change, with τ_R_ dropping from ∼15 ps to ∼6 ps. The values of τ_R_ for core and interfacial water are comparable to those reported for AOT reverse micelles, which is striking considering that AOT has a charged headgroup, whereas phytantriol is neutral and forms distinct mesophase topologies in which the water network varies in both dimensionality and continuity. Notably, in high‐curvature phases ($Ia\bar{3}d$ and *L*_2_), τ_R_ values converge, with interfacial and core water both reaching ∼6 ps, indicating a distorted 3D network where the higher surface curvature compared to the *L*_α_ phase inhibits directional H‐bond formation. This explains the near insensitivity of hydrogen bonds to temperature variations observed in FTIR measurements for the *L*_2_ topology, which features the highest surface curvature and simultaneously disrupts the continuity of the aqueous channels.

Increasing the water content from 10 wt% to 15 wt% affects only the core‐to‐interface ratio (Figure 4g), while reorientation dynamics remain phase‐specific (Figure 4h,i). The τ_R_ value is primarily determined by phase geometry, interface exposure and curvature, which dictates whether water molecules forms 2D or 3D networks. In both the *L*_2_ and $Ia\bar{3}d$ phases, water dynamics are nearly identical and less temperature‐dependent than in the *L*_α_ phase. Although less tightly bound to phytantriol headgroups than in the *L*_α_ phase, the water network remains constrained by the high interfacial curvature characteristic of the *L*_2_ and $Ia\bar{3}d$ phases. This highlights a strong correlation between water dynamics (Figure 4h,i) and structural parameters (Figure 1f). In fact, compared to the 2D confinement of *L*_α_, the 3D water network in the $Ia\bar{3}d$ and *L*_2_ phases reduces the disparity between core and interfacial water. In the *L*_α_ phase, the planar interface enforces long‐lived lipid‐water hydrogen‐bonds that make interfacial water much slower than core water. In contrast, in $Ia\bar{3}d$ and *L*_2_ phases, the curved interfaces and the continuous 3D water networks facilitate exchange and delocalization, making interfacial and core water dynamics more similar.

**Figure 5:**
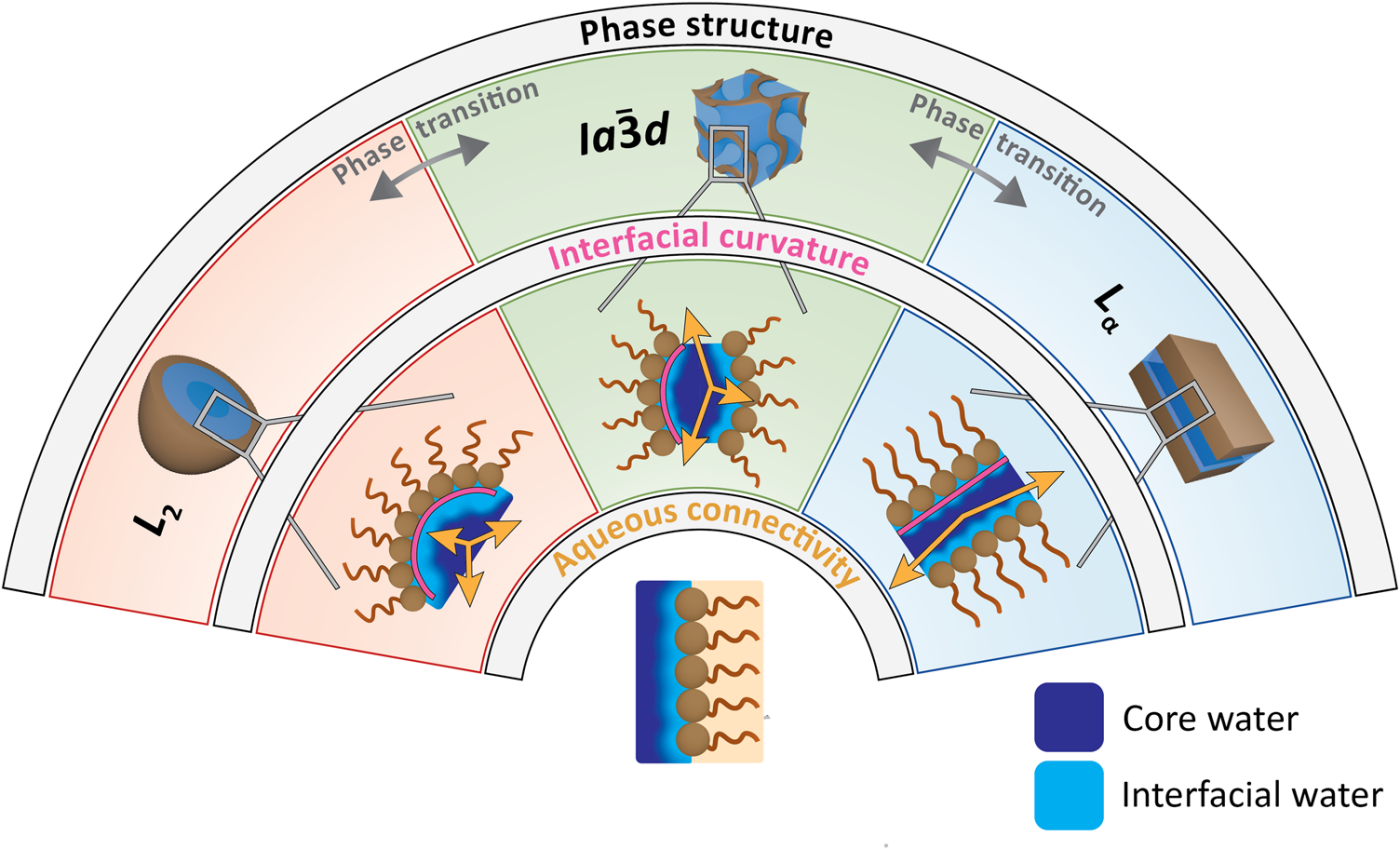
Integrative schematic linking mesophase geometry to water‐network connectivity and dynamics. The outer semicircle shows the three topologies: lamellar phase *L*_α_ (blue), cubic $Ia\bar{3}d$ phase (green), and reverse micellar phase *L*_2_ (red). The middle semicircle highlights the corresponding interfacial curvature (pink) and the dimensionality of the water network (orange arrows). The inner semicircle illustrates core and interfacial water (dark/light blue) and the phytantriol chains (inset). Together, these features summarize how phase geometry controls interfacial curvature, water‐network continuity, and the structural and dynamical signatures probed in this study.

By aligning the outcomes of the experimental techniques employed, we obtain a coherent picture that allows us to illustrate how the phase structure and interfacial curvature of different phytantriol‐water topologies influence the dimensionality and connectivity of the water network under soft confinement, as shown in the integrative schematic in Figure 5.

## Conclusion

Confined water in soft matter is often described as “non‐bulk,” with its structure and dynamics governed not only by confinement but also by interfacial curvature and the continuity of the water network. Using an archaeal‐mimetic phytantriol lipid mesophase system, we can precisely tune the interfacial curvature, the dimensionality of the water network (2D or 3D), and the degree of aqueous continuity across the *L*_α_, $Ia\bar{3}d$, and *L*_2_ phases, thereby directly correlating the confining geometry with the behavior of both the lipid matrix and water molecules. Taken together, the results of all our measurements demonstrate that phase transitions proceed with small, kinetically sensitive enthalpic costs, but trigger discontinuous shifts in hydrogen‐bond populations and a clear increase in chain flexibility upon entering *L*_2_. The electrodynamic response, as retrieved from BDS measurements, bears a distinct geometric imprint: a structure‐sensitive conductivity relaxation accelerates when a 3D continuous water channel forms in $Ia\bar{3}d$ but slows down when continuity is lost in *L*_2_. A dipole‐matrix relaxation is present in the *L*_α_ and $Ia\bar{3}d$ phases but absent in the isotropic *L*_2_ phase. Meanwhile, it is markedly slower in $Ia\bar{3}d$ than in *L*_α_ due to a tighter packing and enhanced interfacial rigidity. Time resolved vibrational spectroscopy highlights that, on picosecond timescales, confined water separates into interfacial and core populations showing different relaxation times (∼2.0 ps versus ∼1.0 ps). The fraction of interfacial water decreases with increasing hydration, and the reorientation time shortens when moving from planar to highly curved surfaces, which affects the connectivity of the water network, rearranging it from 2D to 3D. We observe a strong dependence of water dynamics on phase topology, particularly in terms of interfacial area per lipid headgroup and surface curvature. Notably, 3D connectivity and curved interfaces narrow the interfacial‐core dynamic gap, in sharp contrast to the planar lamellar geometry.

In short, confining geometry governs the chain packing, the hydrogen‐bond network, confined water dynamics, and charge (polaron) transport. These relationships are summarized schematically in Figure 5, which highlights how mesophase geometry determines interfacial curvature, water‐network connectivity, and the resulting dynamical behavior of confined water. The correlation between mesophase geometry and the structure and dynamics of confined water revealed here suggests that geometric control may offer a viable route to tuning diffusion and reactivity within lipidic materials. These insights provide a conceptual basis for guiding the rational design of systems in which confined water mediates key processes, such as molecular transport or enzyme activity.

## Supporting Information

The authors have cited additional references within the Supporting Information.^[^ ^50^, ^51^ ^]^

## Conflict of Interests

The authors declare no conflict of interest.

## Supporting information



Supporting Information

## Data Availability

The data that support the findings of this study are available from the corresponding author upon reasonable request.
