# Supplementary material for: Geometry Controls Confined Water Dynamics in Lipidic Mesophases
Source: Angew Chem Int Ed Engl. 2026 Jan 10;65(7):e22757. doi: 10.1002/anie.202522757 (PMC12887620; doi:10.1002/anie.202522757)
Supplement: Supplementary file 1 — Supporting Information [file ANIE-65-e22757-s001.pdf]

Supporting Information

Geometry Controls Confined Water Dynamics in Lipidic Mesophases

Sara Catalini[a, b, c, †], Matteo Rutsch[a, †], Andrea Lapini[c, d], Barbara Rossi[e], Mariangela Di Donato[c, f], Brenda Bracco[g], Marco Paolantoni[g], and Yang Yao[a*]

[a] Department of Chemistry, University of Basel, Basel, Switzerland

[b] Department of Chemistry “Ugo Schiff”, University of Florence, Florence, Italy

[c] European Laboratory for Non-Linear Spectroscopy, Florence, Italy

[d] Department of Chemical, Life and Environmental Sustainability Sciences, University of Parma, Parma, Italy

[e] Elettra-Sincrotrone Trieste, Trieste, Italy

[f] CNR-ICCOM, Florence, Italy

[g] Department of Chemistry, Biology and Biotechnology, University of Perugia, Perugia, Italy

[†] These authors contributed equally

[*] Corresponding author

Table of Contents

A. Experimental section

1. Samples preparation (page 3)
2. Small-angle X-ray scattering (SAXS) and calculation of structural parameters (page 3-4)
3. Differential scanning calorimetry (DSC) (page 4)
4. Broadband dielectric spectroscopy (BDS) and HN fitting function (pages 4-5)
5. UV Resonance Raman (UVRR) spectroscopy (page 5)
6. Fourier transform infrared (FTIR) spectroscopy (page 6)
7. Pump-probe IR experiment and calculation of water reorientation time (pages 6-8)

B. Supporting Data

Figure S1: 3D SAXS profiles of LMPs (page 9)

Figure S2: Phase transition temperature and enthalpy obtained from DSC (page 9)

Figure S3: Summary of HN fitting parameters obtained from BDS curves (page 10)

Figure S4: Summary of the relaxation times obtained from BDS upon heating (page 11)

Figure S5: Representative BDS curves of the real part of the complex conductivity (page 12)

Figure S6: FTIR and UVRR spectra and data trend as a function of temperature of 5 wt% water LMP (page 13)

Figure S7: FTIR and UVRR spectra and data trend as a function of temperature of 10 wt% water LMP (page 14)

Figure S8. raw experimental transient data (page 15)

Figure S9: temperature dependence of photoproduct yields relative to the instantaneous pump–probe signal (page 15)

C. References

A. Experimental section

1. Samples preparation

Phytantriol (DSM, Heerlen, Netherlands) was used as received. Milli-Q water (18.2 MΩ·cm) served as the aqueous phase. Lipidic mesophase (LMP) samples (total mass 3-5 g) were prepared combining phytantriol and water to desired composition. The mixtures were magnetically stirred under heating with a heat gun for 10 min to ensure homogeneity. After mixing, the samples were left to equilibrate at room temperature overnight, allowing for further structural organization and minimizing trapped air bubbles.

2. Small-angle X-ray scattering (SAXS) and calculation of structural parameters

SAXS experiments were carried out on a Xeuss 3.0 instrument (XENOCS, Grenoble, France), equipped with a Genix 3D X-ray source that generates Cu Kα radiation (λ = 0.154 nm). Scattering data were recorded with a Dectris EIGER2 detector placed 350 mm from the sample. The samples were enclosed in capillaries with a diameter of 1.5 mm. Scattering patterns were acquired over 300 s every 5 °C for a temperature range of 5 – 60 °C using a Peltier temperature control. Prior to data acquisition, samples were equilibrated for 30 min. The mesophase structures were identified by analyzing Bragg reflections at specific relative q-value ratios: 1:2 for the lamellar (L_α) phase; 1:√6:√8:√14:√16:√20:√22 for the cubic $Ia\bar{3}d$ phase; and a single broad peak characteristic of the L_2 phase. Based on the SAXS profiles, the water channel dimensions were calculated for L_α and $Ia\bar{3}d$ phase in the following way:

$$q^* = \frac{2\pi}{a} * \sqrt{h^2 + k^2 + l^2} \tag{1}$$

Where q* is the primary peak position, a is the lattice size, and h,k, and l are the respective Miller indices of L_α and $Ia\bar{3}d$. For the L_α phase, the water lamella thickness d is then calculated using:

$$d = a(1 - \phi_L) \tag{2}$$

with ϕ_L being the lipid volume fraction.

For the cubic $Ia\bar{3}d$ phase, first the lipid length l_L needs to be determined using:

$$\phi_L = 2A_0 \frac{l_L}{a} + \frac{4\pi}{3}\chi\left(\frac{l_L}{a}\right)^3 \tag{3}$$

where χ is the Euler-Poincare characteristic and A_0 the ratio of the area of the minimal surface in the unit cell to the quantity [unit cell volume]$^{2/3}$. The minimal surface parameters of $Ia\bar{3}d$ cubic phase are -8 and 3.091 for χ and A_0, respectively. Finally, the water channel radius r is then calculated using:

$$r = 0.248a - l_L \quad (4)$$

3. Differential scanning calorimetry (DSC)

DSC was used to investigate the phase transition temperature and enthalpy of the LMP samples. Measurements were conducted on a Discovery 25 instrument (TA Instruments, New Castle, USA) equipped with a liquid nitrogen cooling system. Between 4 and 7 mg of each sample was sealed in Tzero™ hermetic aluminum pans (TA Instruments). Prior to measurement, each sample was equilibrated at 60°C for 5 minutes. Subsequently, the temperature was decreased to –15°C at a constant cooling rate of 10 °C/min while monitoring the heat flow. After reaching –15°C, the sample was held at that temperature for 3 minutes before being reheated to 60°C at the same rate. This cooling–heating cycle was repeated twice per sample. The phase transition temperatures and associated enthalpies were analyzed using TRIOS software (TA Instruments) and averaged across the two measurement cycles.

4. Broadband dielectric spectroscopy (BDS) and HN fitting function

BDS was performed over a frequency range of 0.01 Hz to 1 MHz using an Alpha-A High Performance Frequency Analyzer (Novocontrol Technologies GmbH & Co. KG, Montabaur, Germany), equipped with a Quatro temperature control unit and a liquid nitrogen cooling system. For measurements, the samples were placed between two gold-coated electrode plates with a diameter of 20 mm and 40 mm for the upper and lower electrode, respectively. To guarantee consistent spacing between the electrodes, three Teflon spacers (with a thickness of 50 µm) were arranged in a cross-shaped pattern. Dielectric spectra were recorded at temperature intervals of 3°C. Before each measurement, the samples were equilibrated at the target temperature for 3 minutes. The resulting spectra were analyzed in the tan δ representation using Origin Pro 2023. The data were fitted by summing multiple Havriliak–Negami (HN) functions. The HN equation is given by:

$$\varepsilon^*_{HN}(\omega, T) = \varepsilon_\infty(T) + \sum_{k=1}^{l}\left(\frac{\Delta\varepsilon_k(T)}{\left[1+\left(i\omega\tau_{HN,k}(T)\right)^{\alpha_k}\right]^{\gamma_k}} + \frac{\sigma_0(T)}{i\varepsilon_f\omega}\right) \quad (5)$$

Here, k denotes an individual relaxation process, $\Delta\varepsilon$ represents the relaxation strength at temperature T, and $\tau_{HN,k}$ is the characteristic relaxation time. The parameters α_k and γ_k ($0 < \alpha$, $\alpha\gamma \leq 1$) account for the symmetrical and asymmetrical broadening of the relaxation, respectively. ε_∞ is the dielectric permittivity at high frequency limits, σ refers to the dc conductivity, and ε_f is the permittivity of free space. Each dataset was fitted in multiple iterations, using parameter estimates from adjacent temperatures and earlier fitting rounds as starting values. To improve the quality of the fit, selected parameters were fixed or constrained, particularly when parts of the relaxation process extended beyond the accessible frequency range.

For figure S4, relaxation times of the current study were converted from tanδ to ε″ representation through the following relation:

$$\log(\tau_{\varepsilon''}) = \log(\tau_{tan\delta}) + \sqrt{1 + \frac{\Delta\varepsilon}{\varepsilon_h}} \quad (6)$$

Where $\tau_{\varepsilon''}$ and $\tau_{tan\delta}$ are the relaxation times of a relaxation process in ε″ and tanδ representation, respectively, $\Delta\varepsilon$ is the relaxation strength obtained from the fitting shown above (equation 5), and ε_h is the high-frequency limit of the dielectric permittivity.

5. UV Resonance Raman (UVRR) spectroscopy

UVRR spectra were collected using the multi-wavelength UVRR setup available at the BL10.2-IUVS beamline of Elettra Sincrotrone Trieste (Italy). Excitation was provided at 213 nm by an FQSS 213-Q Diode-Pumped Passively Q-Switched Solid-State Laser. Vertically polarized (VV) Raman spectra were acquired in backscattering geometry and analysed using a single-pass Czerny-Turner spectrometer (Trivista 557, Princeton Instruments, 750 mm focal length) equipped with a holographic grating (1800 g/mm) and a UV-optimized CCD camera. Spectrometer calibration was performed using spectroscopic-grade cyclohexane (Sigma-Aldrich). The spectral resolution is 2 cm^{-1}. To prevent photo-damage from prolonged UV exposure, the sample cell was continuously oscillating during data collection. Consistency across repeated measurements confirmed the absence of spectral changes, indicating no photodegradation under UV illumination. All the UVRR spectra were recorded over a temperature range of 10–60 °C in 5 °C increments during heating. After each cycle, the sample was cooled back to 10 °C to verify spectral reversibility. Temperature control was achieved

using a sample holder connected to a thermal bath and a resistive heating system, maintaining a stability of ±0.1°C. Prior to each measurement, samples were equilibrated at the target temperature for 10 minutes. Temperature accuracy was confirmed using an external thermocouple positioned as close as possible to the sample cuvette.

6. Fourier transform infrared (FTIR) spectroscopy

FTIR absorption measurements were performed using a Bruker Invenio R spectrometer at Perugia laboratory (Italy). Spectra were acquired with the Bruker Optics software Opus. Samples were placed in a transmission cell fitted with calcium fluoride (CaF_2) windows. The optical path length was minimized as much as possible without the use of spacers. Once the sample was positioned between the windows, the cell was mounted in a temperature-controlled chamber connected to a thermal bath. Spectra were recorded over the 6000–200 cm^{-1} range, with a resolution of 0.5 cm^{-1}, by averaging 30 scans.

7. Pump-probe IR experiment and calculation of water reorientation time

Samples for the time-resolved IR measurements were prepared following the same protocol described above, except that an HOD mixture was used to obtain an isolated OD oscillator. Samples containing 15% and 10% water included 5% D_2O relative to the total water content, while the sample with 5% water contained 3.5% D_2O. The samples were characterized by recording time-resolved infrared spectra as a function of temperature, ranging from 5°C to approximately 60°C. Transient spectra were acquired using a custom-built cell equipped with CaF_2 windows, thermostatically controlled via a Peltier system, and housed in a jacket maintained at a constant temperature of 20°C by a circulating water bath.

Transient spectra in the mid-infrared (mid-IR) range were acquired at LENS laboratory (Italy). The output of a Ti:Sapphire regenerative amplifier (3.5 mJ per pulse, 1 kHz repetition rate, 40 fs pulse duration) was split into two parts. A 1.3 mJ portion was directed to a TOPAS-800 optical parametric amplifier to generate mid-IR pump pulses centered at 2500 cm^{-1} (250 cm^{-1} FWHM, 70 fs duration, 6 μJ pulse energy), used to selectively excite the OD stretching vibration in HOD molecules. Another 0.4 mJ was sent to a custom-built optical parametric amplifier to produce probe pulses with similar characteristics (centered at 2500 cm^{-1}, 200 cm^{-1} FWHM, 80 fs duration, 1.4 μJ). Both pump and probe pulses were tuned to be resonant with the vibrational transitions of OD oscillators in HOD. The experiment employed a standard pump–probe configuration. The pump pulse, being more energetic, excites the sample and promotes OD oscillators to an excited vibrational state. After a variable delay time (τ), adjustable between 100 fs and 20 ps using a mechanical translation stage, the probe pulse

interrogates the same volume of the sample. It is partially absorbed depending on the population of excited states at that moment. The transient signal is obtained by taking the logarithm of the ratio between the probe intensity with the pump on and the probe intensity with the pump off, yielding a differential spectrum ΔAbs as reported in the scheme 1.

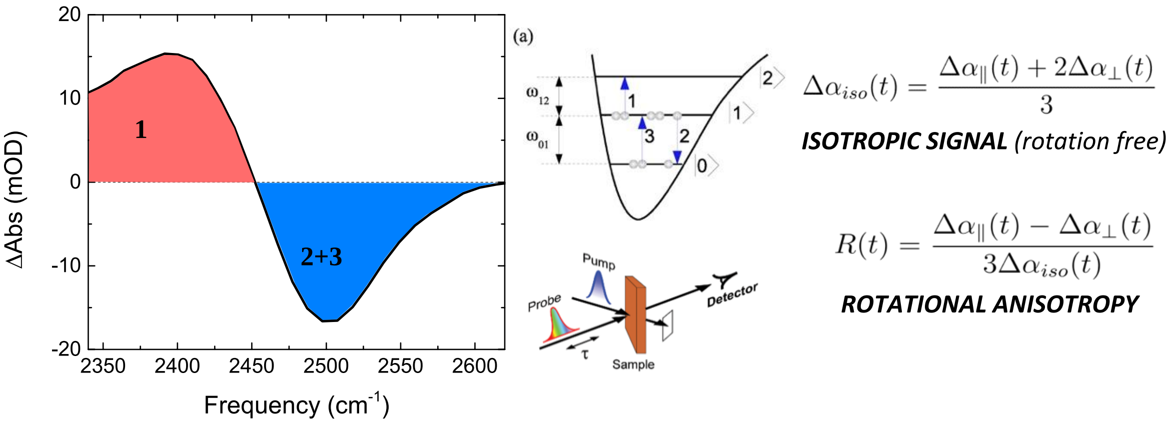

$$\Delta\alpha_{iso}(t) = \frac{\Delta\alpha_{\parallel}(t) + 2\Delta\alpha_{\perp}(t)}{3}$$

$$R(t) = \frac{\Delta\alpha_{\parallel}(t) - \Delta\alpha_{\perp}(t)}{3\Delta\alpha_{iso}(t)}$$

Scheme 1: scheme of pump-probe IR experiment

The negative part of the spectrum (2+3 namely “bleaching” plus “stimulated emission”) is due to less absorption at the frequencies of the ground vibrational state because the pump brought a certain number of molecules to the excited state, plus the stimulated emission caused by the probe pulse from the molecules that are in the excited vibrational level. The positive part (1 namely “excited state absorption”), which is located to lower frequency due to the anharmonicity of the potential, is related to the absorption from the excited vibrational state |1> to state |2>. By recording spectra at various pump–probe delay times, the relaxation dynamics of the vibrationally excited OD stretching mode can be monitored. This provides insight into how quickly the excess vibrational energy introduced by the pump is dissipated.

Most of the energy deposited by the pump pulse is dissipated through vibrational, rotational, and orientational motions. However, a fraction remains as thermal energy, leading to a persistent signal, the thermal residue, on longer timescales. Since the relaxation of this thermal contribution occurs on timescales beyond the detection window of our experiment, it appears as a constant background. The nature of thermal contribution has been investigated so far[1] and related to the increased amount of broken intermolecular hydrogen-bonds following photoexcitation. Taking the transient spectrum at 20 ps delay time, we are quite confident[1-2] to measure the generated photoproduct in thermal equilibrium. The intensity of the differential spectrum of thermal photoproduct is intimately connect with the bulk water molecular spatial and dynamical arrangements and temperature. Our data (see figure 4, main manuscript) clearly

demonstrate that the intensity of the of thermal photoproduct signal, measure ad 20 ps, in respect to the zero-delay time signal depends on the amount of water contained in our sample. Bulk water, namely HOD stretching in H_2O, has a relative signal $n_{water} \approx 0.3$ at room temperature and a slight temperature dependance has been detected, (Figure S8). Hence, the ratio $n_{water} = \Delta Abs_{(t=20\ ps)}/\Delta Abs_{(t=0)}$ has been used to provide an estimate of the residual relative background signal associated with the thermal effect of bulk water, while n_{sample} is the relative signal of thermal residue from water confined in Phy mesophases-cavities. We supposed that the thermal residue signal come from bulk water only, and taking the ratio reported in equation 6 we gave an estimation of the percentage of interfacial water in the sample:

$$\text{Interfacial water } (\%) = 1 - \left(\frac{n_{sample}}{n_{water}}\right) * 100 \qquad (7)$$

The reorientation times, τ_R, of water molecules were obtained from pump-probe measurements. These values were extracted by fitting the rotational anisotropy decay, R(t), with the following mono-exponential function:

$$R(t) = \frac{\Delta\alpha_{//}(t) - \Delta\alpha_{\perp}(t)}{3\Delta\alpha_{iso}(t)} \sim e^{-\frac{t}{\tau_R}} \qquad (8)$$

Rotational anisotropy has been calculated from polarization resolved pump-probe after the correction for thermal contribution described in ref. [2] was applied.

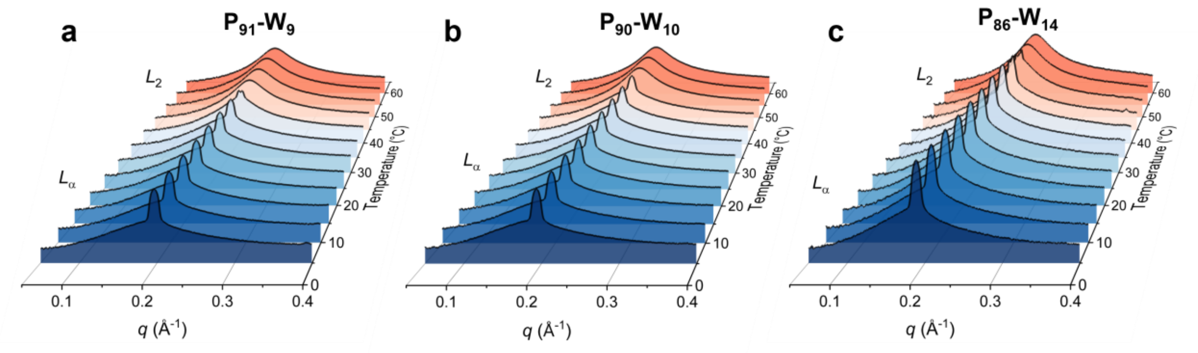

Figure S1. SAXS profiles of LMPs containing 9 wt% (a), 10 wt% (b), and 14 wt% (c) water.

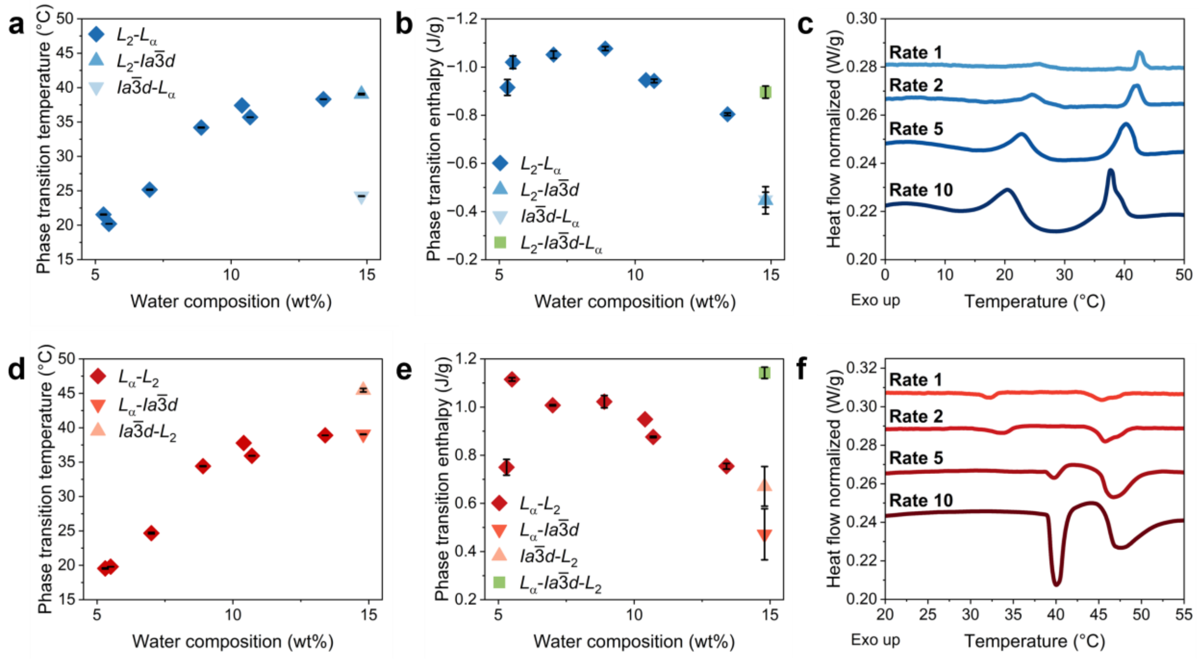

Figure S2. Phase transition temperature (a and d), phase transition enthalpy (b and e), obtained from DSC upon cooling and heating at 10 °C min^{-1}, respectively. Representative DSC curves for the 15 wt% water system at cooling (c) and heating (f) rates of 1 to 10 °C min-1, respectively. Curves were shifted vertically for better visibility. Error bars are obtained from duplicate measurements.

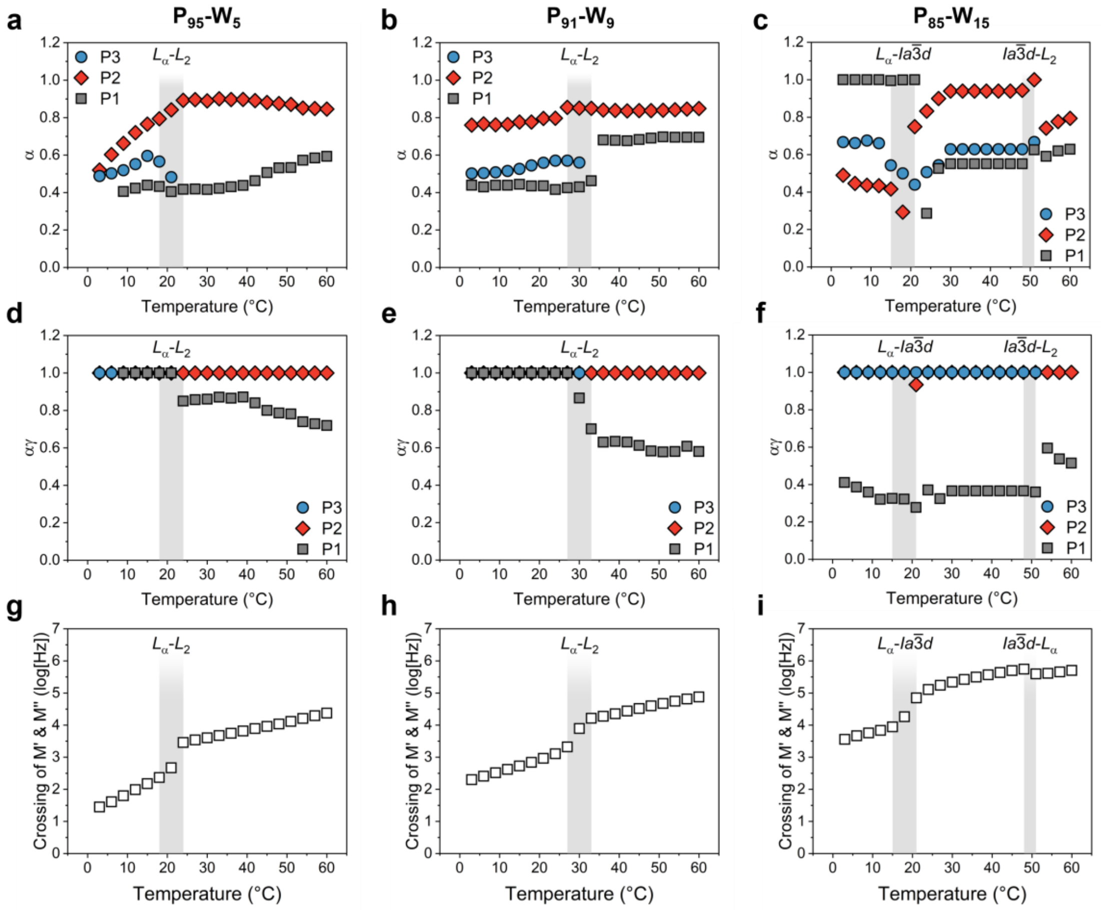

Figure S3. Summary of HN fitting parameters α (a-c), and αγ (d-f), for LMPs with 5 wt%, 9 wt%, and 15 wt% water, respectively, for each individual relaxation process as a function of temperature. Crossing frequencies of the real (M′) and imaginary (M″) part of the complex modulus (M*) as a function of temperature, for LMPs with 5 wt% (g), 9 wt% (h), and 15 wt% (i) water, respectively. The gray areas and labels indicate the phase transitions and corresponding phase symmetries.

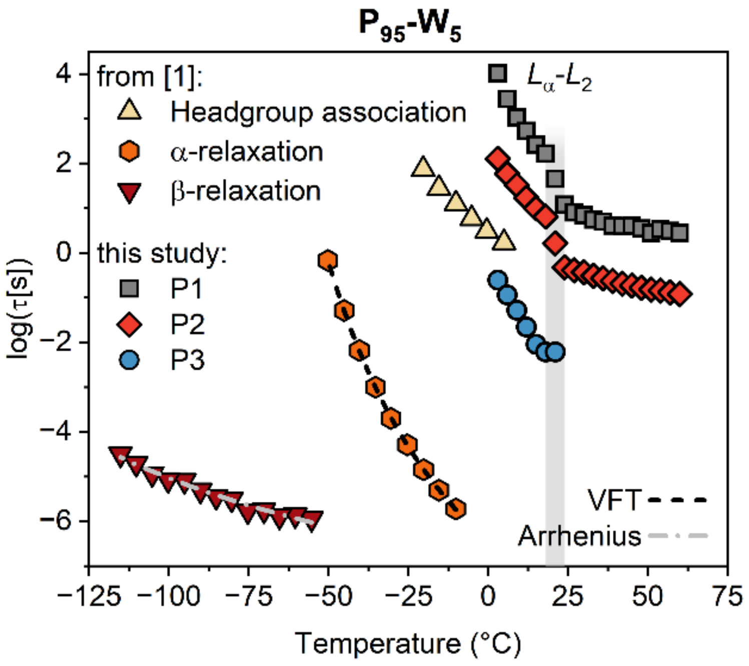

Figure S4. Summary of the relaxation times obtained from BDS upon heating: grey square (P1), red diamonds (P2), blue circles (P3), results of the current study, covering +3 to +60 °C, relaxation times were converted using equation 6 (SI); yellow up triangles (headgroup association), orange hexagons (α-relaxation), red down triangles (β-relaxation), obtained from Yao et al., 2021[3], covering -120 to +5 °C. The dashed and dash-dotted lines are fits according to the Vogel-Fulcher-Tammann (VFT) and Arrhenius equations, respectively.

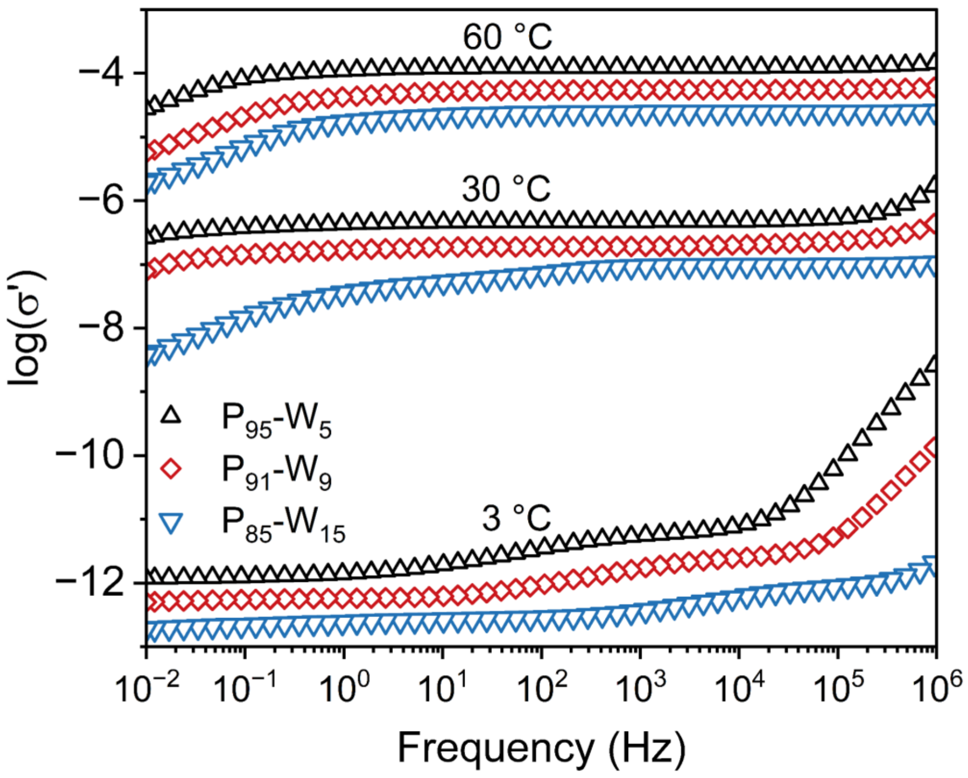

Figure S5. Representative BDS curves of the real part of the complex conductivity, σ′, at 3 °C, 30 °C, and 60 °C, for LMPs containing 5 wt%, 9 wt%, and 15 wt% water. Data points were shifted vertically for better visibility. The drop at the low frequency end indicates electrode polarization.

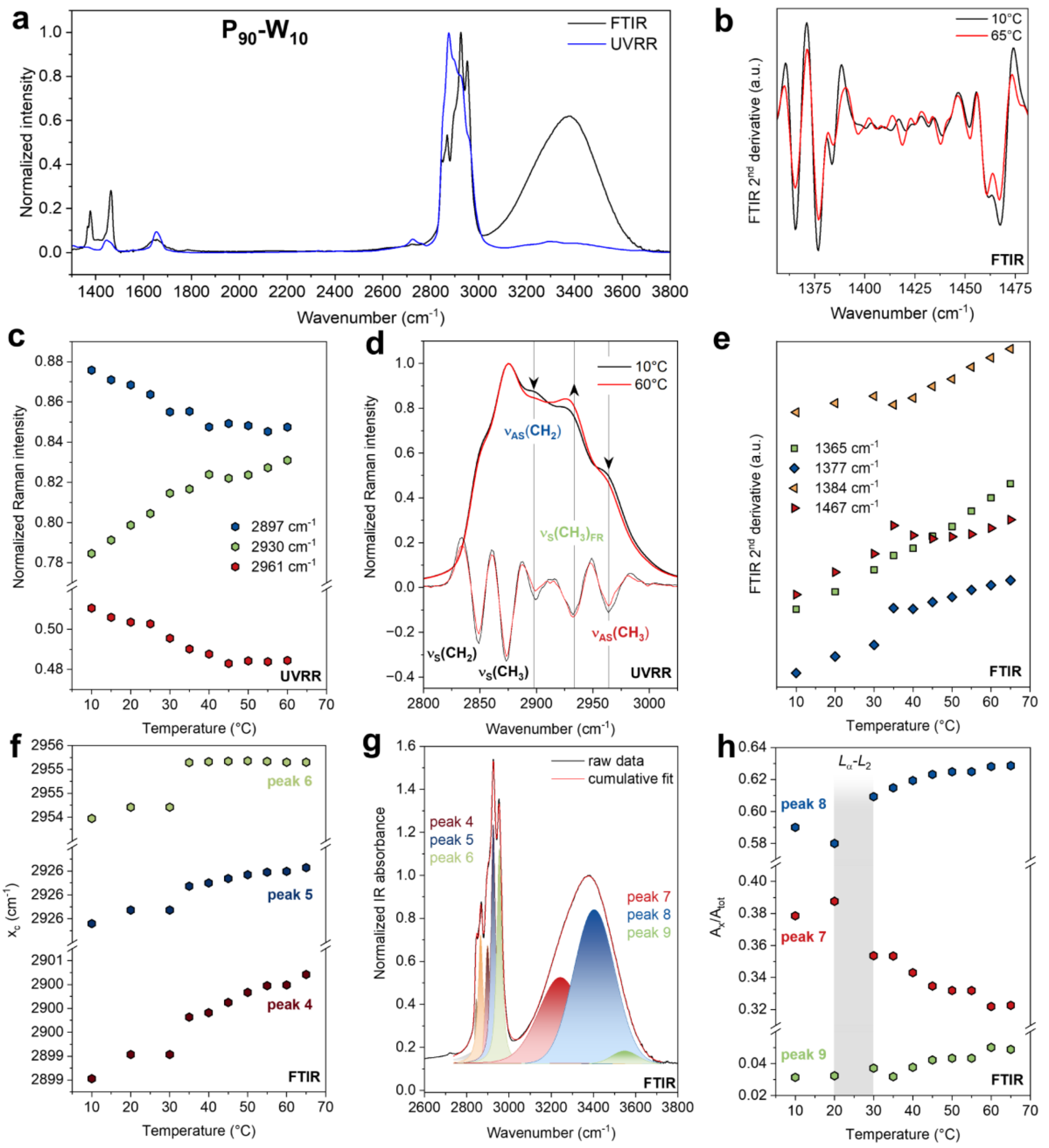

Figure S6. (a) FTIR and UVRR spectra of 5 wt% water LMP recorded at 10 °C. (b) Second derivative of the FTIR spectra at 10 °C and 60 °C. (c) Temperature-dependent Raman intensities of the peaks indicated by arrows in panel (d). (d) UVRR spectra at 10 °C and 60 °C, with corresponding second derivatives to better resolve and assign spectral components. (e) Temperature trends of FTIR spectra second derivative peak intensities. (f) Temperature dependence of the frequency positions of vibrational signals in the lipid region, obtained from FTIR spectral fitting. (g) Fitting example of the FTIR spectrum in the 2600–3800 cm^{-1} region at 10 °C. (h) Temperature-dependent fractions of highly ordered, partially disrupted and weakly stabilized OH stretching components s derived om fitting the FTIR spectra of 5 wt% water LMP. The grey shaded areas indicate the phase transitions with the corresponding phase symmetries.

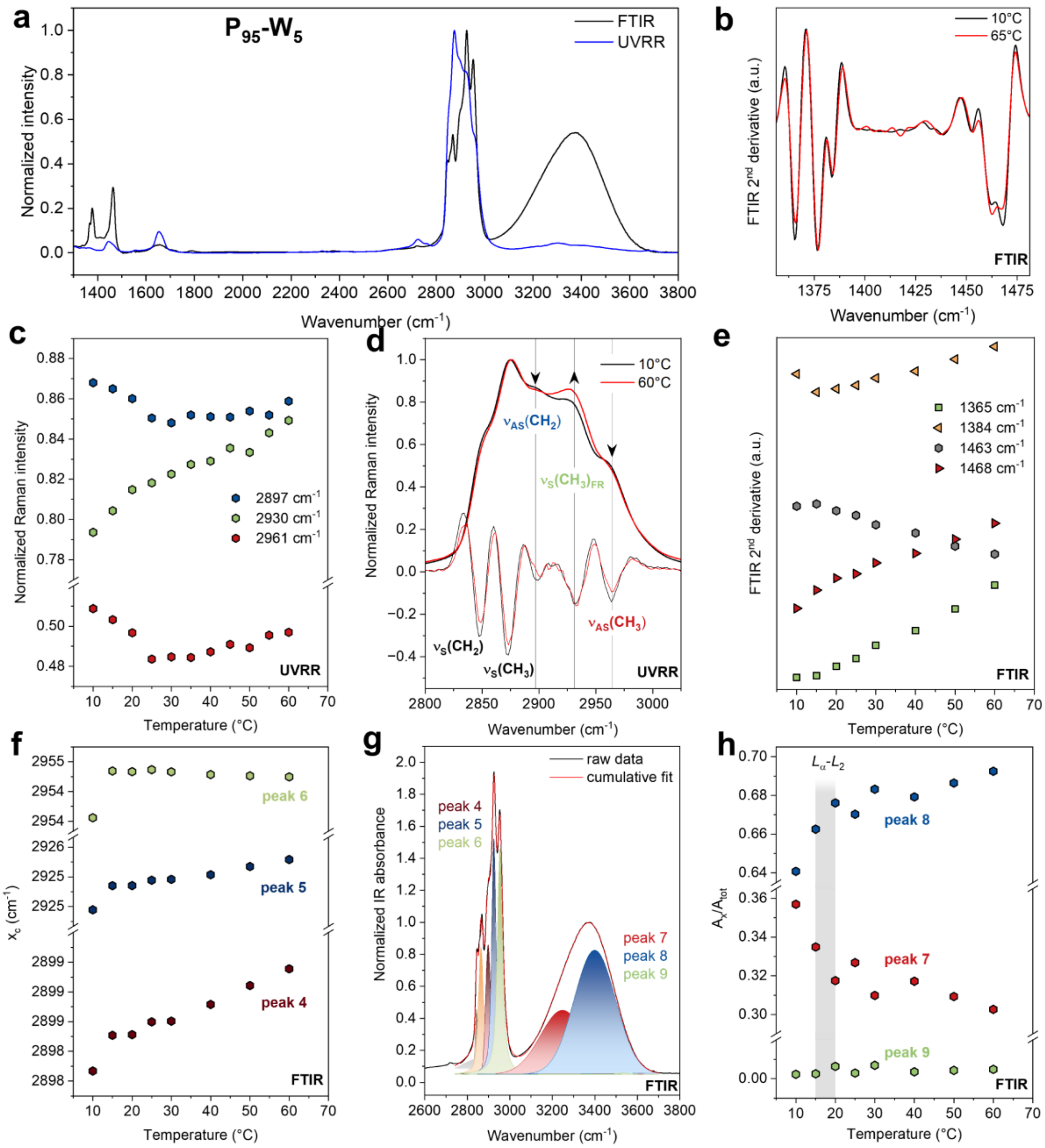

Figure S7. (a) FTIR and UVRR spectra of 10 wt% water LMP recorded at 10 °C. (b) Second derivative of the FTIR spectra at 10 °C and 65 °C. (c) Temperature-dependent Raman intensities of the peaks indicated by arrows in panel (d). (d) UVRR spectra at 10 °C and 60 °C, with corresponding second derivatives to better resolve and assign spectral components. (e) Temperature trends of FTIR spectra second derivative peak intensities. (f) Temperature dependence of the frequency positions of vibrational signals in the lipid region, obtained from FTIR spectral fitting. (g) Fitting example of the FTIR spectrum in the 2600–3800 cm^{-1} region at 10 °C. (h) Temperature-dependent fractions of highly ordered, partially disrupted and weakly stabilized OH stretching components derived om fitting the FTIR spectra of 10 wt% water LMP. The grey shaded areas indicate the phase transitions with the corresponding phase symmetries.

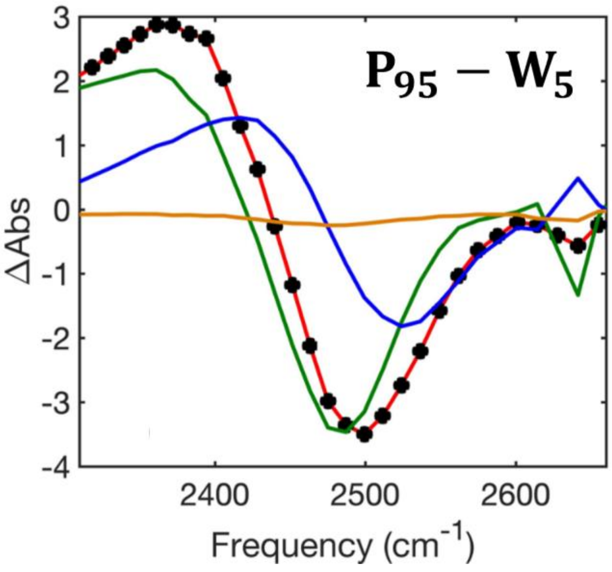

Figure S8. raw experimental transient data (black points) of 5 wt% water, overlapped with the fitting function (red curve). The red curve is composed by two components one at higher frequency (blue curve) and one with a lower frequency (green curve). The orange curve is the residue of the fit.

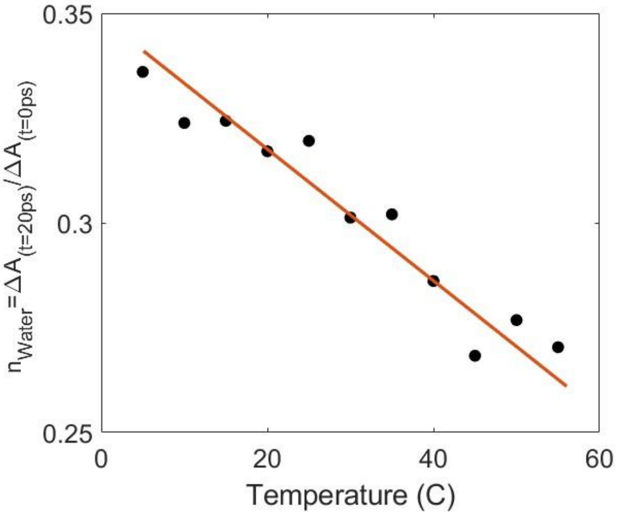

Figure S9. Temperature dependence of thermal photoproduct signal intensity (pump-probe measured at 20 ps) in respect with the pump-probe signal measured at zero-time delay.

References

[1] T. Steinel, J. B. Asbury, J. R. Zheng, M. D. Fayer, *Journal of Physical Chemistry A* **2004**, *108*, 10957-10964.

[2] Y. L. A. Rezus, H. J. Bakker, *J Chem Phys* **2005**, *123*.

[3] Y. Yao, T. Zhou, R. Färber, U. Grossner, G. Floudas, R. Mezzenga, *Nat Nanotechnol* **2021**, *16*, 802-+.
